# Association Between Visceral Adiposity Index and Insulin Resistance: A Cross-Sectional Study Based on US Adults

**DOI:** 10.3389/fendo.2022.921067

**Published:** 2022-07-22

**Authors:** Kai Jiang, Hong Luan, Xiaolu Pu, Mingxiang Wang, Jiahui Yin, Rongpeng Gong

**Affiliations:** ^1^ Department of Cardiovascular Medicine, The Bozhou Hospital Affiliated to Anhui Medical University, Bozhou, China; ^2^ Department of Cardiovascular Medicine, People’s Hospital of Ningxia Hui Autonomous Region, YinChuan, China; ^3^ Medical College of Qinghai University, Xining, China; ^4^ College of Traditional Chinese Medicine, Shandong University of Traditional Chinese Medicine, Jinan, China

**Keywords:** cross-sectional study, american adult, VAI, IR, NHANES

## Abstract

**Background:**

Visceral obesity index (VAI) is an empirical mathematical model used to evaluate the distribution and function of fat. Some studies have shown that VAI may be associated with the development of insulin resistance. In view of the differences in insulin resistance among different ethnic groups, this study attempts to analyze the special relationship between VAI and insulin resistance in American adults.

**Methods:**

We conducted a cross-sectional study through NHANES database. A total of 27309 patients over the age of 18 from the United States took part in the survey. It was divided into two groups: the IR-positive group and the IR-negative group. The association of VAI with IR was evaluated by logistic regression analyses mainly, including univariate analysis, multivariate regression analysis, curve fitting analysis and subgroup analysis.

**Results:**

The results showed that in the full-adjusted model, there is a strong positive association between VAI level and insulin resistance (OR: 1.28 (1.2~1.37), P<0.001) and there is a threshold effect.

**Conclusions:**

This study suggests that higher VAI levels are associated with insulin resistance. VAI index may be used as a predictor of insulin resistance.

## Background

Insulin resistance (IR) is a pathological condition caused by genetic and environmental factors, in which insulin promotes the decrease of glucose uptake and utilization rate, as well as the body’s decreased responsiveness and sensitivity to the physiological action of insulin (1, 2). It is the pathological basis of type 2 diabetes (2–6). Recently, the prevalence of diabetes has risen rapidly in all developing and developed countries (7), and type2 diabetes is the most common type of diabetes, accounting for approximately 90% of all people with diabetes (8, 9). It is estimated that by 2030, the number of people with type 2 diabetes will reach 439 million (10). Current studies have demonstrated that understanding IR is important for developing prevention measures and determining optimal treatment. Unfortunately, the methods available to determine IR (such as pancreatic suppression tests, high insulin-normal blood sugar tuse and glucose digestion and the minimum model of metabolism) are complex and expensive, so they apply only to small-scale studies (11–13). In view of these characteristics, it is necessary to find alternative parameters that are low-cost and convenient. Visceral obesity index (VAI) is a gender-specific mathematical index that has been proposed to assess fat distribution and function. VAI is estimated with the use of simple anthropometric [body mass index (BMI) and waist circumference (WC) and biochemical triglycerides (TG) and high-density lipoprotein cholesterol (HDL-C)] parameters (14). VAI is generally considered a as a marker of adipose tissue dysfunction. As a simple technique, the index has been widely accepted for epidemiological or clinical research. Some recent studies have indicated that visceral obesity index was also correlated with IR (15–19). Homeostasis model assessment of high insulin-normal glucose (HIEG) forcers and IR (HOMA-IR), which is the gold standard and common method for IR assessment. Given the small sample size of previous studies, the ethnic specificity of IR, and the unexplored relationship between VAI and IR in these studies, it is necessary to conduct new, large-sample studies to understand VAI and IR.

Visceral obesity index (VAI) is an empirical mathematical model that has been proposed to assess fat distribution and function. It is a sex-specific index based on simple anthropometry (BMI and WC) and metabolic parameters (TG and HDL-C). Interesting results have been produced by the application of VAI in populations of patients with endocrine diseases with varying degrees of cardiometabolic risk, such as acromegaly, polycystic ovary syndrome, type 2 diabetes, and prolactinoma (20–24). This has led to the hypothesis that VAI can be regarded as a marker of adipose tissue dysfunction. Some recent studies have indicated that the VAI can be successfully used to detect the distribution and function of visceral fat, IR and increased cardiometabolic risk (25, 26). In a study conducted by Jablonowska-Lietz et al. (18) VAI index was also significantly correlated with glucose, insulin, HOMA-IR, and visceral adipose tissue predicted by bioimpedance analysis. Stepien et al. also suggested a positive correlation between IR and VAI in obese patients (15). In another study, Borruel et al. (19) reported that VAI levels were more strongly correlated with serum insulin levels and HOMA-IR than WC and BMI levels. Because of its simple technique, the index has been widely accepted for epidemiological or clinical research. Given the small sample size of previous studies, the ethnic specificity of IR, and the unexplored relationship between VAI and IR in these studies, it is necessary to conduct new, large-sample studies to understand VAI and IR.

Therefore, we explored the relationship between VAI index and IR in a larger and more representative sample of various ethnic groups in the United States.

## Methods

### Data Sources

It was a large cross-sectional study using data from the National Institutes of Health National Health and Nutrition Examination Survey (NHANES) database for 11 cycles (1999-2020). The NHANES program is a multiagency collaboration aimed at improving the health of Americans, with a focus on diet, detailed elsewhere (27). NHANES used a multi-stage stratified probability design in a sample population to obtain a nationally representative sample of non-institutionalized civilians in the United States. Data from these samples consisted of demographic informatics data, dietary data, body measurement data, laboratory data and questionnaire data. In this study, data from 11 cycles were standardized and combined with fasting weights as recommended by the National Center for Health Statistics (NCHS).

### Study Design and Participants

This study was designed to be cross-sectional. The target independent variable was the participant’s VAI at the time of testing, and the target dependent variable was whether the participant was diagnosed with IR at the time of testing. Simultaneously, the occurrence of IR was divided into two groups, including 11936 patients in the positive group of IR and 15373 patients in the negative group of IR.

**Figure 1:**
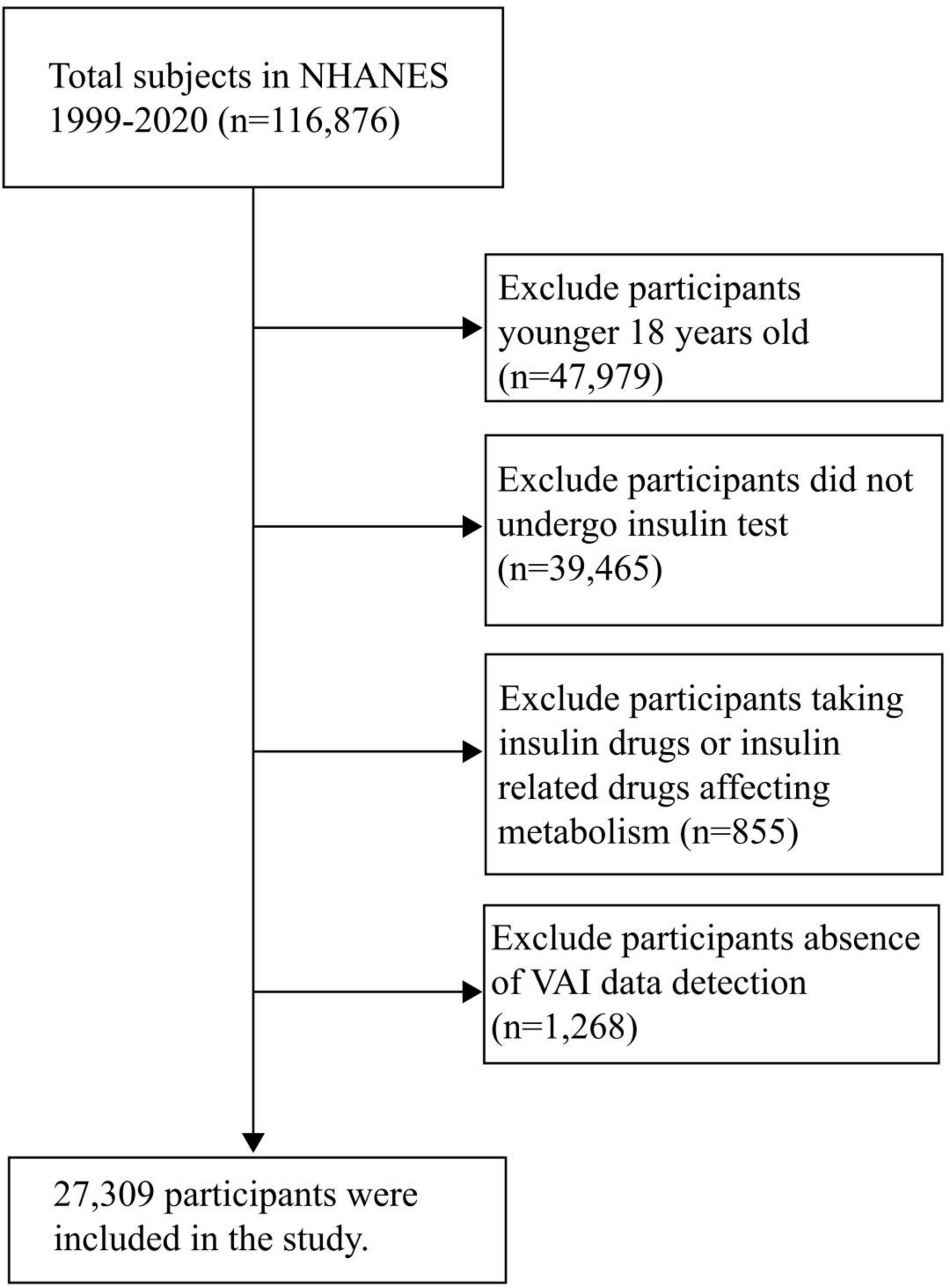
Flowchart of patient selection.

A total of 116,876 participants were included in NHANES 1999-2020, and 27,309 were included in the final analysis. Other participants were excluded for the following reasons: 1. Participants younger than 18 years old (n= 47979); 2. Participants who did not undergo insulin test (n= 39465); 3. Participants who were taking insulin drugs or insulin related drugs affecting metabolism (n=855); 4. Participants lack VAI data detection (n= 1268) (**Figure 1**).

### Data Collection

All data were collected and recorded by uniformly trained investigators. The data used in this study included demographics (age, sex, race, education level, etc.), anthropometry (WC, BMI, etc.), health-related behaviors (smoking, drinking, etc.), and biochemical indicators (TG, VAI, etc.). Basic information was immediately collated by investigators, and biochemical samples were stored and managed scientifically before being sent to the University of Minnesota laboratory and the University of Missouri-Columbia for testing and analysis.

#### Measurement of VAI

VAI is a simple clinical index that integrates anthropometric data and metabolic parameters, and can better assess visceral fat. It was calculated as follows: Man = [WC(cm)/39.68 + (1.88 ×BMI)]×(TG(mmol/L)/1.03)×(1.31/HDL(mmol/L)); Women = [WC (cm)/36.58 + 1.89 × (BMI)] × (TG (mmol/L)/0.81) × (1.52/HDL (mmol/L)). BMI is calculated based on height and weight. Height was measured using electronic Sports Measurements (Seca Ltd, Medical Scales and Measurement Systems, Birmingham, UK) with an accuracy of millimetres. Weight was measured by researchers using a digital Scale (Toledo Scale; Mettler-toledo, LLC, Columbus,OH, USA) and convert pounds to kilograms when the measurement is complete. The formula is BMI =weight (kg)/height (m^2^). WC was measured using electronic Sports Measurements (Seca Ltd, Medical Scales and Measurement Systems, Birmingham, UK) with an accuracy of millimetres. HDL was measured by the Magnesium sulfate/glucan method and TG was measured based on the Wahlefeld method. Both HDL and TG were measured in the University of Minnesota laboratory. Please refer to the official NHANES website for more detailed information.

#### Measurement of Insulin Resistance

**Figure 2:**
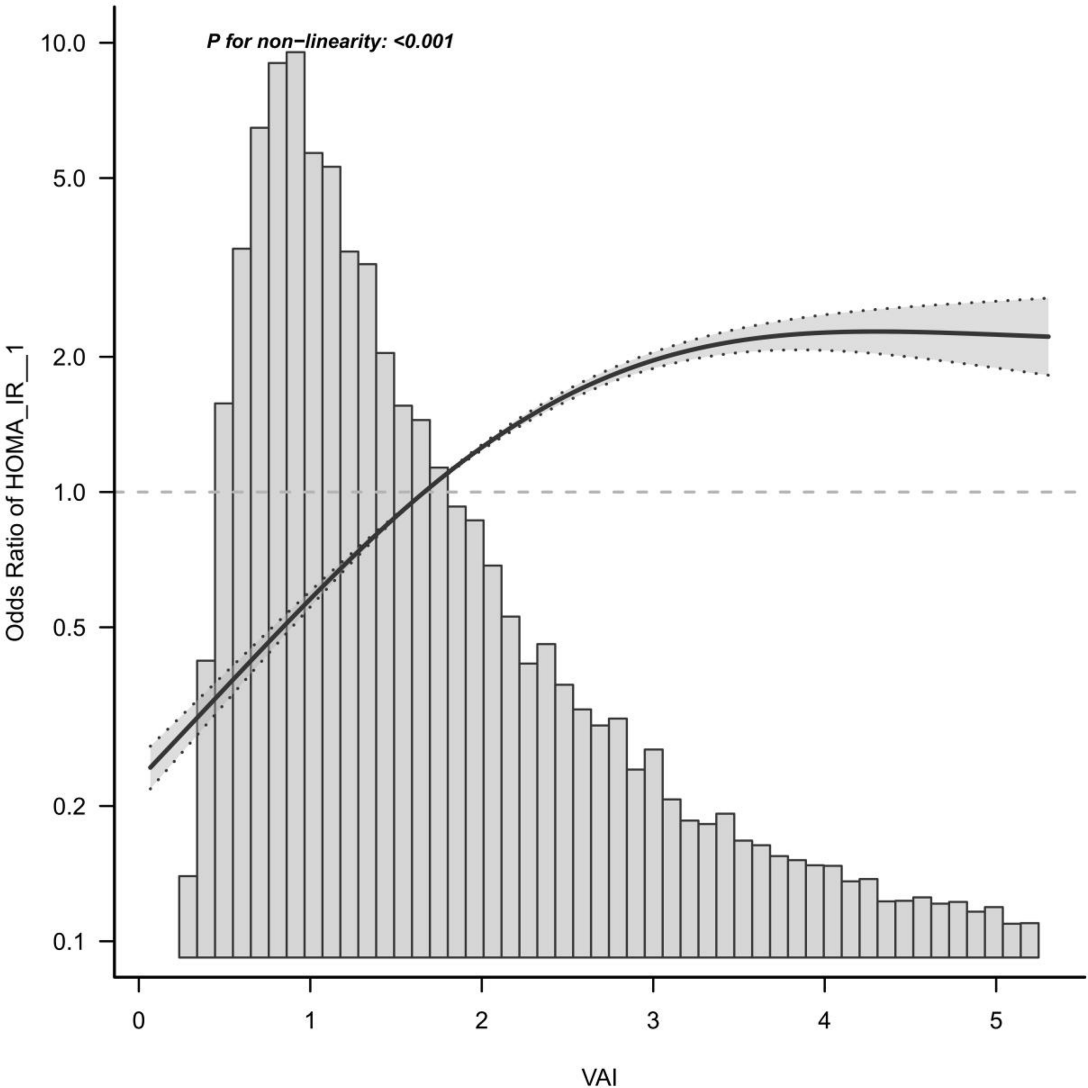
Curve fitting analysis of VIA and IR.

HOMA-IR is recognized by many experts as a good indicator of IR. The formula was fasting blood glucose (FPG, mmol/L) × fasting insulin (FINS, μU/mL)/22.5 (**Figure 2**). We followed previous studies that defined homA-IR index ≥ 2.73 as positive insulin resistance, and < 2.73 as negative insulin resistance. Fasting blood glucose was measured by hexokinase (HK) method, and fasting insulin was measured by insulin radioimmunoassay. Both blood sugar and insulin measurements were tested at the University of Missouri-Columbia. Enter the official NHANES website for more detailed information.

#### Definitions of Other Variables

Age: Adults 18 and older in the United States.

Sex: man, woman.

Race: Includes Mexican Americans, non-Hispanic whites, non-Hispanic Blacks, other Hispanics, and other races.

Education: not graduated from high school, high school, graduate and above.

Smoking: current smokers, former smokers and never smokers. Participants were considered current smokers if they had smoked 100 or more cigarettes in the past and reported smoking several days or daily at the time of the interview. Participants who had smoked fewer than 100 cigarettes in the past but did not currently smoke were considered former smokers. Participants who had fewer than 100 cigarettes in their past were considered nonsmokers. Alcohol consumption: Includes both drinkers and non-drinkers.

Alcohol consumption (minus 1 point for alcoholics) is defined as more than one drink per day for women and more than two drinks per day for men, according to the US Department of Health and Human Services/US Department of Agriculture Dietary Guidelines for Americans 2015-2020.

Hypertension: Including those with hypertension and normal blood pressure, the diagnostic criteria are SBP higher than 140mmHg and/or DBP higher than 90mmHg.

Diabetes: Including diabetic patients and people with normal blood glucose.Diabetes is diagnosed if one of the following conditions is met: (1) fasting blood glucose ≥ 7.0mmol/L, (2) OGTT ≥ 11.1mmol/L, (3) doctor’s diagnosis, (4) self-report diabetes or taking diabetes drugs.

#### Laboratory Quality Control

NHANES Quality Control and Quality Assurance Protocols (QA/QC) meet the requirements of the Clinical Laboratory Improvement Act 1988. Detailed QA/QC instructions are discussed in the NHANES LPM.

### Statistical Methods

All data were analyzed using version R 4.1.2, with continuous variables represented by a detailed sample description, an average confidence interval of 95%, and categorical variables represented by counting and weighted percentages. The normal distribution is described by median and standard deviation, and the skewness distribution is based on median and quartiles. Continuous variables were compared between groups using mann-Whitney U test or Student T test based on distribution normality. P < 0.05 (bilateral) was considered statistically significant. The choice of the covariate was based on the previous literature, international standards and related clinical experience of synthetically considering may influence factors of IR and visceral fat index, including sex, age, race, smoking, drinking, education degree, diabetes, hypertension. In order to maximize statistical efficiency and minimize bias, multiple imputation was used to fill in covariates within the range of missing and extreme values. In addition, sensitivity analysis was performed to observe if the new complete data were significantly varied from the original data. However, these studies revealed that there was no significant difference between the data after multiple interpolation and the original data (P > 0.05). Therefore, all the results of our multivariate analysis are based on the data set after multiple interpolation according to Rubin’s criterion. In this study, four multivariable logistics regression models were established to analyze the relationship between VAI and IR in U.S. adults. In order to verify whether the results are inapplicable to the current population, we divided the results into groups according to sex, age, race, smoking, alcohol consumption, BMI, education level, diabetes, hypertension, etc., to observe whether the results are stable in each subgroup. Additionally, trend test was carried out to transform the VAI from continuous variable to categorical variable, and a smooth fitting curve and threshold effect model were constructed to ensure the stability of results.

## Results

### Description of Basic Information About Participants

A total of 27309 participants were included in this study, including 11936 insulin resistance positive and 15373 insulin resistance negatives. The mean age and standard deviation of insulin resistance positive participants (49.0 ± 18.4) were higher than those of insulin resistance negative participants (45.9 ± 19.1), and the difference was significant (p < 0.001). There were 6,012 men (50.4%) slightly higher than 5,924 women (49.6%). In the racial distribution of the United States, non-Hispanic whites taken up the highest proportion of 4,555 cases (38.2%), while non-Hispanic blacks accounted for the lowest proportion of 1,145 cases (9.6%). BMI (32.2 vs 25.9), WC (106.9cm vs 90.7cm), TG (1.5mmol/L vs 1.0mmol/L), alanine transaminase (ALT) (23.0mmol/L vs 18.0mmol/L), γ-glutamyl transpeptadase (GGT) (24.0mmol/L vs 17.0mmol/L), blood urea nitrogen (BUN) (4.6mmol/L vs 4.3mmol/L), VAI (2.0 vs 1.1) were higher than those in the insulin resistance negative group. Compared to insulin resistance negative group, insulin resistance positive group have higher BMI (32.2 vs 25.9), WC (106.9cm vs 90.7cm), TG (1.5mmol/L vs 1.0mmol/L), alanine transaminase (ALT) (23.0mmol/L vs 18.0mmol/L), γ-glutamyl transpeptidase (GGT) (24.0mmol/L vs 17.0mmol/L), blood urea nitrogen (BUN) (4.6mmol/L vs 4.3mmol/L), VAI (2.0 vs 1.1). The difference was significant (P < 0.001). In contrast, HDL (1.4mmol/L vs 1.2mmol/L) and totalbilirubin (TBIL) (12.0mmol/L vs 10.3mmol/L) in the insulin resistance negative group were higher than those in the insulin resistance positive group, and the difference was significantly (P < 0.001). In addition, there were differences in education level, smoking, alcohol consumption, hypertension and diabetes between the two groups (all P < 0.001) (**Table 1**).

**Table 1:** Basic crowd information description.

Variables	Total (n = 27309)	IR-negative (n = 15373)	IR-positive (n = 11936)	P-value
Age, Mean ± SD	47.2 ± 18.9	45.9 ± 19.1	49.0 ± 18.4	< 0.001
Gender, n (%)				< 0.001
male	13299 (48.7)	7287 (47.4)	6012 (50.4)	
female	14010 (51.3)	8086 (52.6)	5924 (49.6)	
Race, n (%)				< 0.001
Mexican American	4937 (18.1)	2350 (15.3)	2587 (21.7)	
Other Hispanic	5677 (20.8)	3107 (20.2)	2570 (21.5)	
Non-Hispanic white	11562 (42.3)	7007 (45.6)	4555 (38.2)	
Non-Hispanic black	2333 (8.5)	1188 (7.7)	1145 (9.6)	
Other races	2800 (10.3)	1721 (11.2)	1079 (9)	
Education, n (%)				< 0.001
poorly educated	6503 (23.8)	3290 (21.4)	3213 (26.9)	
Moderately educated	5877 (21.5)	3212 (20.9)	2665 (22.3)	
highly educated	13053 (47.8)	7717 (50.2)	5336 (44.7)	
NA	1876 (6.9)	1154 (7.5)	722 (6)	
BMI, Mean ± SD	28.6 ± 6.7	25.9 ± 4.9	32.2 ± 6.9	< 0.001
Waist, Mean ± SD	97.8 ± 16.2	90.7 ± 13.0	106.9 ± 15.5	< 0.001
Smoke, n (%)				< 0.001
never smoking	14303 (52.4)	8034 (52.3)	6269 (52.5)	
former smokers	6337 (23.2)	3256 (21.2)	3081 (25.8)	
Current smoker	5300 (19.4)	3238 (21.1)	2062 (17.3)	
NA	1369 (5.0)	845 (5.5)	524 (4.4)	
Alcohol use, n (%)				< 0.001
no	16185 (59.3)	8775 (57.1)	7410 (62.1)	
yes	11124 (40.7)	6598 (42.9)	4526 (37.9)	
Hypertension, n (%)				< 0.001
no	17302 (63.4)	10953 (71.3)	6349 (53.2)	
yes	10003 (36.6)	4418 (28.7)	5585 (46.8)	
DM, n (%)				< 0.001
no	4343 (15.9)	1053 (6.8)	3290 (27.6)	
yes	22966 (84.1)	14320 (93.2)	8646 (72.4)	
HDL, Median (IQR)	1.3 (1.1, 1.6)	1.4 (1.2, 1.7)	1.2 (1.0, 1.4)	< 0.001
TG, Median (IQR)	1.2 (0.8, 1.7)	1.0 (0.7, 1.4)	1.5 (1.0, 2.1)	< 0.001
ALT, Median (IQR)	20.0 (15.0, 28.0)	18.0 (14.0, 24.0)	23.0 (17.0, 32.0)	< 0.001
AST, Median (IQR)	22.0 (18.0, 27.0)	22.0 (18.0, 26.0)	22.0 (19.0, 28.0)	< 0.001
GGT, Median (IQR)	20.0 (14.0, 30.0)	17.0 (13.0, 25.0)	24.0 (17.0, 36.0)	< 0.001
TBIL, Median (IQR)	10.3 (8.6, 13.7)	12.0 (8.6, 15.4)	10.3 (6.8, 13.7)	< 0.001
BUN, Median (IQR)	4.6 (3.6, 5.7)	4.3 (3.6, 5.4)	4.6 (3.6, 5.7)	< 0.001
VAI, Median (IQR)	1.4 (0.9, 2.4)	1.1 (0.7, 1.8)	2.0 (1.3, 3.3)	< 0.001

BMI, Body Mass Index; DM, diabetes mellitus; HDL, high-density lipoprotein; TG, triglyceride; ALT, Alanine aminotransferase; AST, Aspartate aminotransferase; GGT, γ-glutamyl transpeptadase; TBIL, total bilirubin; BUN, blood urea nitrogen; VAI, visceral obesity index.

### Univariate Analysis

We analyzed correlations between age, sex, race, education, BMI, WC, smoking, alcohol consumption, and several biochemical markers and IR in the U.S. population. We found that age was positively correlated with IR, and the effect value OR and 95% confidence interval were 1.01 (1.01, 1.02), respectively. Compared with men, women had a lower risk of IR, with an effect value and 95%CI of 0.76 (0.65, 0.90), respectively. Among ethnic groups, non-Hispanic whites had a lower incidence of IR, with an effect value of 0.57 (95%CI, 0.42, 0.76). The incidence of IR was lower in those with higher education than in those with lower education and secondary education, and the effect value and 95%CI were 0.68 (0.54, 0.85), respectively. Compared with non-smokers, former smokers had a higher risk of IR, with an effect value and 95%CI of 1.24 (1.02, 1.51), respectively. Current smokers had a low incidence of IR, with an effect value and 95%CI of 0.81 (0.65, 1.00), respectively. Compared with non-drinkers, drinkers had a lower risk of IR, with an effect value and 95%CI of 0.75 (0.63, 0.89), respectively. Meanwhile, we found that BMI, WC and some biochemical indicators were positively correlated with the occurrence of IR, including TG, ALT, GGT and VAI (**Table 2**).

**Table 2:** Univariate analysis for IR. (weight).

Variable	OR (95%CI)	P-value
Age	1.01 (1.01~1.02)	<0.001
Gender		
male	1	
female	0.76 (0.65~0.9)	0.001
Race		
Mexican American	1	
Other Hispanic	0.75 (0.52~1.07)	0.116
Non-Hispanic white	0.57 (0.42~0.76)	<0.001
Non-Hispanic black	0.82 (0.53~1.25)	0.359
Other races	0.61 (0.4~0.92)	0.017
Education		
poorly educated	1	
Moderately educated	0.86 (0.66~1.1)	0.23
highly educated	0.68 (0.54~0.85)	0.001
BMI	1.25 (1.22~1.27)	<0.001
Waist	1.1 (1.09~1.11)	<0.001
Smoke		
never smoking	1	
former smokers	1.24 (1.02~1.51)	0.029
Current smoker	0.81 (0.65~1)	0.052
Alcohol1 use		
no	1	
yes	0.75 (0.63~0.89)	0.001
HDL	0.13 (0.1~0.17)	<0.001
TG	2.11 (1.89~2.36)	<0.001
ALT	1.03 (1.02~1.04)	<0.001
AST	1.01 (1~1.01)	0.02
GGT	1.01 (1.01~1.02)	<0.001
TBIL	0.96 (0.94~0.98)	<0.001
BUN	1.08 (1.03~1.13)	0.001
VAI	1.6 (1.5~1.71)	<0.001

BMI, Body Mass Index; DM, diabetes mellitus; HDL, high-density lipoprotein; TG, triglyceride; ALT, Alanine aminotransferase; AST, Aspartate aminotransferase; GGT, γ-glutamyl transpeptadase; TBIL, total bilirubin; BUN, blood urea nitrogen.

### Multi-Factor Analysis

We established four logistic regression models to analyze the relationship between VAI and IR, the effect value of the model can be interpreted as with the increase of VAI, the probability of IR increases correspondingly. For example, in model 1 (Unadjusted model), the incidence of IR increased by 60% with each increase of variance of VAI, and the effect value OR and 95%CI were 1.60 (1.50, 1.71), respectively. Model 2 was adjusted according to population characteristics (age, gender and race), and its effect value OR and 95%CI were 1.62 (1.51, 1.73), respectively. The effect value OR and 95%CI of model 3 were 1.29 (1.20, 1.38), respectively. The effect value OR and 95%CI of model 4 were 1.28 (1.20, 1.37), respectively. The results of model 3 and model 4 were similar, indicating that the adjustment strategy of model 4 was sufficient. Collectively, VAI is independently positively correlated with the occurrence of IR, which can be used as a predictor of IR. Further, in order to ensure the stability of the results, the trend test was carried out in this study. The VAI was transformed from continuous variable to categorical variable and grouped into four levels according to the quartiles of VAI. Q1 was taken as the reference, the incidence of VAI and IR represented a monotonically increasing trend in all models (All P for trend < 0.001). This suggests that VAI is positively correlated with the occurrence of IR and the results are stable (**Table 3**).

**Table 3:** The association between VAI and IR in a multiple logistics regression model.

Variable	n(%)	Model 1		Model 2		Model 3		Model 4	
		OR (95%CI)	*P*-value	OR (95%CI)	*P*-value	OR (95%CI)	*P*-value	OR (95%CI)	*P*-value
VAI	27309	1.6 (1.5~1.71)	<0.001	1.62 (1.51~1.73)	<0.001	1.29 (1.2~1.38)	<0.001	1.28 (1.2~1.37)	<0.001
VAI group									
Q1	6827	1(Ref)		1(Ref)		1(Ref)		1(Ref)	
Q2	6827	2.55 (1.91~3.4)	<0.001	2.69 (2.01~3.61)	<0.001	1.87 (1.34~2.61)	<0.001	1.79 (1.28~2.5)	0.001
Q3	6827	5.52 (4.18~7.29)	<0.001	6.01 (4.51~8.03)	<0.001	3.06 (2.2~4.27)	<0.001	2.79 (2~3.91)	<0.001
Q4	6828	12.27 (9.25~16.28)	<0.001	13.73 (10.22~18.45)	<0.001	5.73 (4.07~8.06)	<0.001	5.03 (3.56~7.12)	<0.001
P for trend	27309	2.27 (2.09~2.47)	<0.001	2.35 (2.15~2.57)	<0.001	1.77 (1.6~1.97)	<0.001	1.69 (1.52~1.88)	<0.001

Model 1: non-adjusted.

Model 2: adjutesd age, gender, race.

Model 3: adjutesd age, gender, race, education, smoke, Alcohol use, diabetes, hypertension.

Model 3: adjutesd age, gender, race, education, smoke, Alcohol use, diabetes, hypertension, ALT, AST, GGT, BUN.

### Subgroup Analysis

**Figure 3:**
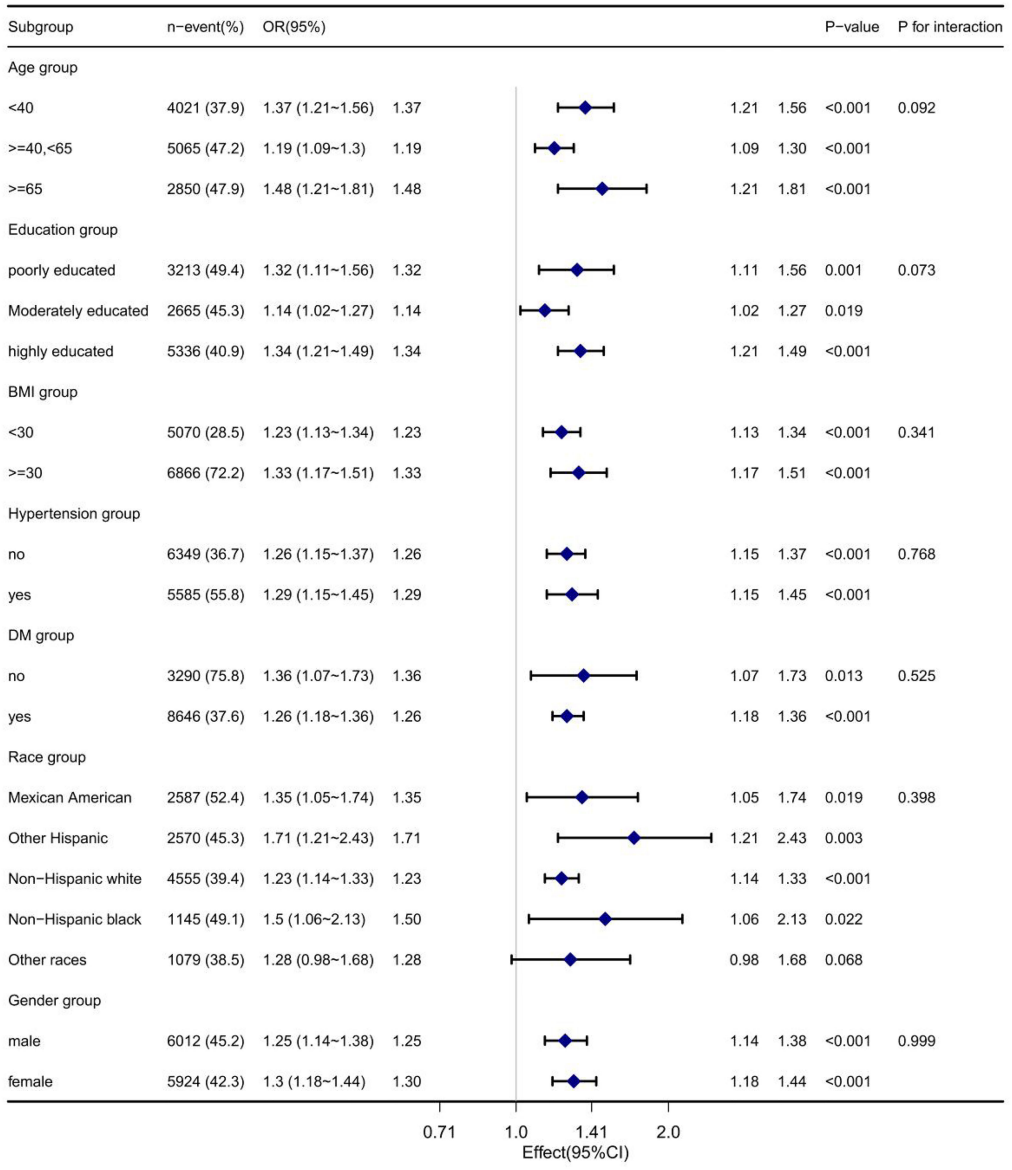
Subgroup analyses of the association between VIA and IR.

We did a subgroup analysis by age, education, BMI, and so on to observe if the results were not applicable to the current population. As shown in **Figure 3**, the relationship between VAI and insulin resistance remained stable across all subgroups, including age, education, BMI, diabetes, race, and sex.

### Curve Fitting and Threshold Effect Analysis

Here, a smooth curve fitting diagram was drawn to visually describe the relationship between VAI and IR, and the linear relationship was tested. As shown in **Figure 3**, the correlation between VAI and insulin resistance is sloping, and P for non-linearity < 0.001, which indicates that the correlation between VAI and IR cannot be assessed by a single logistics regression equation. Therefore, the threshold effect is analyzed. As shown in **Table 4**, there was a threshold effect between VAI and insulin resistance, with an inflection point of 1.92. After adjustment according to model 4, the effect value OR and 95%CI on the left side of inflection point were 2.647 (2.406, 2.913), respectively. The OR and 95%CI on the right side of inflection point were 1.327 (1.245, 1.414), respectively. Moreover, the effect values on the left and right sides of the inflection point are different, and P for Likelihood Ratio test is less than 0.001.

**Table 4:** Threshold effect analysis.

Outcome	OR (95%)	*P*-vale
Break Point	1.92 (1.883,1.956)	NA
slope1	2.647 (2.406~2.913)	<0.001
slope2	1.327 (1.245~1.414)	<0.001
Likelihood Ratio test	–	<0.001
Non-linear Test*1	–	<0.001
Non-linear Test*2	–	<0.001

Covariates are adjusted using variables in Model 4.

## Discussion

This is a large cross-sectional study using 11 cycles of data from the NHANES database to investigate the association between VAI index and IR in America adults. The results revealed that VAI was independently positively correlated with the incidence of IR among all ethnic groups in the United States. and could be used as one of the predictors of IR.

In current clinical practice, heterogeneity in insulin measurement between laboratories in various countries is ubiquitous, which clearly raise the cost and accuracy of determining IR. Therefore, a simple and convenient IR determination system is extremely vital for clinical purposes. VAI determined by WC, BMI, fasting TG, and HDL-C has been established and is considered a more comprehensive IR indicator. VAI exhibit simple, accessible, cheap and at the forefront of glucose and insulin, unlike gold standard HIEG forceps and other IR replacement markers which are complex, time-consuming and costly and dependent on glucose and insulin. The study confirmed a significant association between VAI and HIEG clamps and insulin resistance (16, 17), indicating the great potential of VAI as a useful indicator of accurate IR levels.

In a previous study on the relationship between VAI and IR, Randria Arisoa et al. (28)reported that VAI was positively correlated with HOMA-IR in non-diabetic Germans (β = 0.42, P < 0.0001). A prospective cohort study conducted by Ji et al. (29) also found that very high VAI was the main risk factor for the rise of HOMA-IR in Chinese adults. Similarly, Stepien et al. (15) also revealed a positive correlation between IR and VAI levels in obese patients. In another study, Borruel et al. (19) suggested that VAI levels were more strongly correlated with serum insulin levels and HOMA-IR than WC and BMI levels. In the Framingham Heart Study, Preis et al. (30) reported that visceral adipose tissue and abdominal subcutaneous adipose tissue were positively correlated with IR, and that visceral adipose tissue was more strongly correlated with IR than abdominal subcutaneous adipose tissue.

The possible mechanism of the relationship between VAI and insulin resistance is as follows:Visceral adipocytes secrete adipose-specific cytokines, such as leptin and adiponectin, as well as inflammatory cytokines (tumor necrosis factor-α and interleukin 6), which increase IR (31, 32). Macrophages accumulate in visceral adipose tissue and release these inflammatory cytokines, including tumor necrosis factor-α and interleukin-6, which impair insulin sensitivity (33). Excess adipose tissue can promote inflammation by increasing the level of resistin or tumor necrosis factor-α, thus increasing IR (34, 35). Reduced adiponectin levels associated with excess adipose tissue can exacerbate metabolic disorders and IR (36).

Several limitations of this study should be mentioned. (1) The time span is long, and there are differences in the methods used to determine IR. However, more samples are allowed to be included according to our current approach, and the detection method is very clear. Considering that all samples are tested by professional testing institutions, this effect can be ignored. (2) Certain deviations are inevitable in cross-sectional studies. We will conduct cohort studies in the future when conditions permit. (3) This study assessed IR using HOMA-IR index. The “gold standard” method (e.g., hyperinsulin-normal glucose clamp and hyperglycemic clamp tests) is more accurate than the HOMA index in measuring IR. Although HOMA indices are not “gold standard” methods, they may be more suitable in large epidemiological studies (37). Because of the limited sample size, we can’t analyze special populations and other ethnic groups. Therefore, whether this result is applicable to special populations and populations in other countries needs further research. We will collect these samples for analysis in future studies to cover the deficiencies of this study.

Despite these limitations, there are some significant advantages. (1) The data used is large and generalized. (2) NHANES is an internationally recognized high-quality database exhibit comprehensive and reliable, which greatly enriches research data. (3) Our study used curve fitting and threshold effect analysis to further analyze the relationship between VAI and IR. (4) A more advanced multiple interpolation method was adopted to deal with missing data, and the sensitivity of interpolation data was analyzed. The results indicated that the interpolated data not much differed from the original data, which makes our results more convincing.

## Conclusion

This study explored the relationship between VAI and IR in depth. The association between VAI and IR was a threshold effect after adjustment for potential confounders. At the right of the inflection point, the association between VAI and IR was weakened, which has great significance for the further development of predictive models of IR in the U.S. population. However, the causal relationship between VAI and IR cannot be determined owing to the cross-sectional nature of this study, and a large number of prospective studies are still needed to investigate.

## Data Availability Statement

Publicly available datasets were analyzed in this study. This data can be found here: https://www.cdc.gov/nchs/nhanes/.

## Author Contributions

RG and KJ conceived the idea. RG, KJ, and MW wrote the manuscript. RG read through and corrected the manuscript. KJ is the first author. RG and JY are the corresponding authors of this paper. All authors contributed to the article and approved the submitted version.

## Funding

This study was Supported by the Young Teachers Research Fund of Anhui University of Science and Technology (QN2018128).

## Conflict of Interest

The authors declare that the research was conducted in the absence of any commercial or financial relationships that could be construed as a potential conflict of interest.

## Publisher’s Note

All claims expressed in this article are solely those of the authors and do not necessarily represent those of their affiliated organizations, or those of the publisher, the editors and the reviewers. Any product that may be evaluated in this article, or claim that may be made by its manufacturer, is not guaranteed or endorsed by the publisher.

## Abbreviations

VAI, visceral obesity index; WC, waist circumference; TG, triglyceride; HDL-C, density lipoprotein cholesterol; ALT, alanine transaminase; GGT, γ-glutamyl transpeptadase.
